# Hepatocellular Carcinoma in Two Patients with Autoimmune Hepatitis, A Single Center Experience and Review of the Literature

**DOI:** 10.5812/hepatmon.7957

**Published:** 2013-04-17

**Authors:** Bita Geramizadeh, Saman Nikeghbalian, Alireza Shamsaifar, Kurosh Kazemi, Seyed Ali MalekHosseini

**Affiliations:** 1 Transplant Research Center, Department of Pathology, Shiraz Medical School, Shiraz University of Medical Sciences, Shiraz, IR Iran; 2 Department of Pathology, Shiraz Medical School, Shiraz University of Medical Sciences, Shiraz, IR Iran; 3 Department of Surgery, Shiraz Medical School, Shiraz University of Medical Sciences, Shiraz, IR Iran

**Keywords:** Hepatitis, Autoimmune, Carcinoma, Hepatocellular

**Figure 1:**
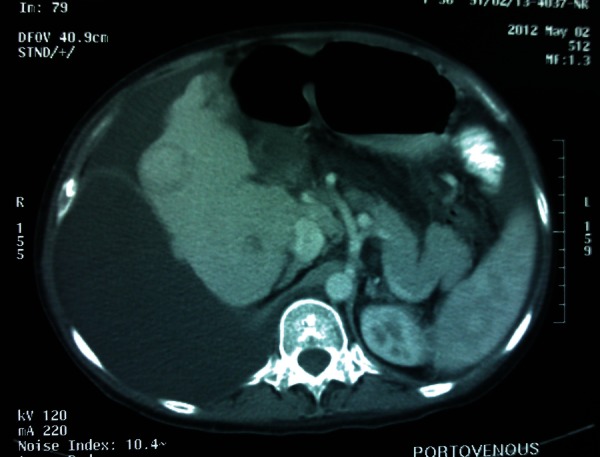
CT Scan of the Liver Shows a Well-Defined Hypoechoic Lesion in Right Lobe, 3X1.9X1 cm.

Dear Editor,

The coincidence risk of autoimmune hepatitis (AIH) and hepatocellular carcinoma (HCC) in the absence of viral hepatitis is extremely low and most of the previous reports have been isolated case reports (1). Meanwhile, most of the previous cases have been reported before HCV screening and it is believed that some of them were secondary to HCV co-pathogenesis (2). Therefore, HCC is a rare sequel of AIH, and an incidence of about 1% has been reported from different geographic areas (3). Herein we report our experience in two female patients aged 35 and 39 with AIH and cirrhosis, who affected by HCC. Case No. 1 was diagnosed as AIH in 2005. She was admitted in the liver transplant list. Alpha-fetoprotein (AFP) level was 4.4 ng/mL (normal < 5 ng/mL). Routine work up for the liver transplantation was started. Magnetic resonance imaging (MRI) demonstrated a contrast enhancing lesion with the diameter of 3.5 cm (Figure 1). Liver biopsy showed well differentiated HCC. She was successfully transplanted, the explanted liver showed cirrhosis and a well-defined 3.5 cm single nodule in the right lobe. She had an uneventful postoperative period and now after two years, she is in a good condition without HCC recurrence. Case No. 2 was diagnosed as AIH in 2003, She was admitted in the liver transplant list. She was considered in the liver transplant waiting list. Alpha-fetoprotein (AFP) level was 14.2 ng/mL. Magnetic resonance imaging (MRI) demonstrated a contrast enhancing lesion with the diameter of 3.5 cm. Liver biopsy showed well differentiated HCC. She was successfully transplanted, the explanted liver showed cirrhosis and two well-defined nodules in the right lobe, 3X3X2.5 cm and 1X1x1 cm. Both of the nodules were well differentiated hepatocellular carcinoma and hilum free. Now after 3 months, she is in good condition. Both of the patients had negative viral markers for hepatitis B and C. In contrast to hepatitis B and C, the reported incidence of HCC in AIH related cirrhosis is very low (4). In our center during 18 years, 231 patients with cirrhosis secondary to AIH have been transplanted (160 females and 71 male patients). Among these 231 patients, only two cases of HCC have been detected (0.09%). The reported incidence of HCC in the patients with AIH related cirrhosis differs in various countries and the highest has been reported from Japan, i.e. 1.9%-3.3% (2, 5). The pathogenesis of development of HCC in the patients with cirrhosis secondary to AIH has not been definitely identified, however an impaired immune function which is characteristic of this disease can be involved in carcinogenesis (2). Also the persistent chronic low grade hepatocellular inflammation and regenerative activity in AIH may predispose to malignancy (1). There are different reported risk factors for the development of HCC in the patients with AIH related HCC, such as absence of ALT normalization (2). This problem has been present in both of our patients, i.e. none of them had completely normal ALT during their disease. The duration of disease before the development of HCC has been about 10 years in previous reports and it has been claimed that the rate of HCC onset increases at 10 years or longer after the diagnosis of AIH (5). This duration was seven and nine years in our patients. Other reported risk factors have been steatosis, non-alcoholic fatty liver disease, and obesity (2). One of the most challenging issues in the patients with AIH is that whether regular screening strategy for HCC is necessary or not. In the studies from Japan, most of the reports believe on routine cancer screening and surveillance programs; However it is controversial and still there is no consensus in this area (5). As a conclusion, although HCC is rare in the Iranian patients with cirrhosis related AIH, it should be considered as a possible occurrence and routine follow up by sonography and AFP level may be justified (5).
